# Interdisciplinary Collaborative Virtual Reality Planning for Chest Wall Resection and Reconstruction for Sarcoma and Other Large Chest Wall Malignancies Enhanced by Automated AI Segmentation: A Retrospective Comparative Analysis

**DOI:** 10.1055/a-2727-1789

**Published:** 2025-11-24

**Authors:** Philipp Schnorr, Benedetta Bedetti, Jan Wynands, Sebastian Koob, Hruy Menghesha, Jens Buermann, Donatas Zalepugas, Jan Arensmeyer, Joachim Schmidt, Philipp Feodorovici

**Affiliations:** 1 155843 Department of Thoracic Surgery, Helios Hospital Bonn Rhein-Sieg, Bonn, Germany; 2 39062 Departement of Thoracic Surgery, University Hospital Bonn, Bonn, Germany; 3 39062 Department of General, Visceral, Vascular and Transplant Surgery, University Hospital Bonn, Bonn, Germany; 4 39062 Bonn Surgical Technology Center (BOSTER), University Hospital Bonn, Bonn, Germany; 5 39062 Department of Orthopedics and Trauma Surgery, University Hospital Bonn, Bonn, Germany

**Keywords:** virtual reality surgical planning, chest wall resection and reconstruction, interdisciplinary surgical planning, chirurgische Planung mittels Virtual Reality, Thoraxwand-Resektion und Rekonstruktion, interdisziplinäre Resektionsplanung

## Abstract

**Purpose:**

Oncologic chest wall resection and reconstruction present significant surgical challenges due to the complex interplay of anatomical and physiological factors. Ensuring adequate oncologic margins while preserving structural integrity and function is essential for optimal oncological and physiological patient outcomes. Advanced visualization technologies such as virtual reality (VR) are being increasingly investigated for surgical use cases because of their ability to provide a comprehensive and immersive representation of anatomical structures, thereby enhancing preoperative planning and, potentially, intraoperative guidance. The goal of this study is to establish a streamlined workflow using state-of-the-art technology to optimize surgical planning and potentially improve patient outcomes in the complex field of chest wall reconstruction.

**Patients/Materials and Methods:**

Eight cases of complex chest wall resection were retrospectively analyzed using the “Medical Imaging XR” VR platform with AI-driven anatomical auto segmentation. An interdisciplinary team of surgeons collaboratively planned the surgical procedures in VR, and predicted parameters such as resection extent, defect dimensions, and reconstruction strategies. These were then quantitatively compared with actual intraoperative findings. User experience was assessed with the User Experience Questionnaire (UEQ), workspace perception ratings, and Simulator Sickness Questionnaire (SSQ).

**Results:**

In 3 cases (37.5%), the actual resection exceeded the VR-predicted extent due to underestimated tumor infiltration. Planning exceeded resection in 50% of cases by up to 24% and one case (12.5%) showed a large overestimation in VR. UEQ scores showed high hedonic quality (Stimulation = 2.19, Novelty = 2.69) and positive pragmatic usability (Efficiency = 1.13, Dependability = 1.63). Workspace perception was favorable (mean 4.9/6), and cybersickness remained low.

**Conclusion:**

AI-enhanced VR planning enables interdisciplinary collaboration and can improve spatial understanding in complex chest wall surgery. Although it facilitates structured preoperative planning and communication, it should be viewed as a complementary tool to select the surgical strategy rather than as a definitive predictor of the extent of resection. Limitations in imaging resolution and segmentation accuracy can lead to under- or overestimation of tumor boundaries. Further development and clinical validation are necessary to determine its full impact on surgical planning quality and outcomes.

## Zusammenfassung

**Hintergrund:**

Resektion und Rekonstruktion der Brustwand bei Tumorerkrankungen stellen aufgrund des komplexen Zusammenspiels anatomischer und physiologischer Faktoren eine erhebliche chirurgische Herausforderung dar. Für ein optimales onkologisches und funktionelles Ergebnis ist es entscheidend, onkologische Sicherheitsabstände zu gewährleisten und dabei strukturelle Integrität und Funktion zu erhalten. Fortschrittliche Visualisierungstechnologien, wie die virtuelle Realität (VR), werden zunehmend chirurgisch eingesetzt, da sie eine umfassende und immersive Darstellung anatomischer Strukturen ermöglichen. Ziel dieser Studie war es, mithilfe modernster Technologie einen optimierten Arbeitsablauf für die präoperative Planung zu etablieren und so potenziell das Outcome von Brustwandrekonstruktionen zu verbessern.

**Patienten/Materialien und Methoden:**

Acht Fälle komplexer Brustwandresektionen wurden retrospektiv mit der VR-Plattform „Medical Imaging XR“ und KI-gestützter anatomischer Autosegmentierung analysiert. Ein interdisziplinäres Team aus Chirurg*innen plante die Eingriffe gemeinsam in der VR-Umgebung und prognostizierte Parameter wie Resektionsausmaß, Defektgrößen und Rekonstruktionsstrategien. Diese wurden anschließend quantitativ mit den tatsächlichen intraoperativen Befunden verglichen. Die Nutzererfahrung wurde mittels verschiedener Scores erhoben.

**Ergebnisse:**

In 37,5% der Fälle überschritt die tatsächliche Resektion den in VR vorhergesagten Umfang, 12,5% zeigten eine hohe Überschätzung. In 50% zeigte sich eine dezente Überschätzung von bis zu 24%. Die Nutzererfahrungen zeigten eine hohe Bewertung von Stimulation und Neuheit sowie eine positive pragmatische Nutzbarkeit auf. Die Arbeitsbereichswahrnehmung war positiv (Ø = 4,9/6), Cybersickness niedrig.

**Schlussfolgerung:**

Die KI-gestützte VR-Planung unterstützt die interdisziplinäre Zusammenarbeit und kann das räumliche Verständnis bei komplexen Thoraxwandeingriffen verbessern. Sie erleichtert die strukturierte präoperative Planung und Kommunikation, sollte jedoch mehr als ergänzendes Werkzeug zur Wahl der Operationsstrategie und nicht als verlässlicher Prädiktor für das Resektionsausmaß verstanden werden. Einschränkungen bei der Bildauflösung und Segmentierungsgenauigkeit können zu einer Unter- oder Überschätzung der Tumorgrenzen führen. Technische Weiterentwicklungen und klinische Validierung sind notwendig, um den vollen Einfluss auf Planungsqualität und Outcome zu bestimmen.

## Abbreviations

2D: Two-dimensional
3D: Three-dimensional
AI: Artificial intelligence
CT: Computed tomography
GPU: Graphics processing unit
HMD: Head-mounted display
PACS: Picture Archiving and Communication System
SSQ: Simulator-Sickness Questionnaire
UEQ: User-Experience Questionnaire
VR: Virtual reality
XR: Extended reality

## Introduction

Due to the complex interplay of anatomical and physiological factors chest wall resection and reconstruction remain a significant challenge. Ensuring adequate oncologic margins while preserving structural integrity and function is essential for optimal patient outcomes 1 . In cases of larger tumors, especially sarcomas involving soft tissue and bone structures of the chest wall, a multidisciplinary approach is advised for both resection and reconstruction 2 3 . These complex procedures typically require not only an experienced thoracic surgeon, but also a plastic surgeon, especially when reconstruction involves extensive skin defects or the need to fill dead space using muscle flaps 3 . Sarcomas and metastatic disease are the most frequent chest wall malignancies. Sarcomas are complex entities and therapeutic strategies should be discussed in a multidisciplinary tumor board in a certified sarcoma center 4 . Furthermore, when resection of adjacent structures is necessary, the involvement of an experienced onco-orthopedic surgeon is crucial during preoperative planning to ensure comprehensive and effective surgical management 5 .

Traditional surgical planning often relies on two-dimensional (2D) visualization modalities that may inadequately represent complex three-dimensional (3D) anatomical relationships. This limitation can contribute to intraoperative difficulties, especially during the reconstruction of large defects, and may potentially lead to suboptimal physiological and oncologic outcomes. Advanced visualization technologies such as extended reality (XR) and its sub-term virtual reality (VR) are being increasingly investigated for surgical use cases due to their ability to provide a comprehensive and immersive representation of anatomical structures, thereby enhancing preoperative planning and, potentially, intraoperative guidance 6 7 8 . Recent developments in collaborative VR platforms have further expanded the opportunities for surgical decision-making by enabling interdisciplinary collaboration 9 . Previous research has highlighted the benefits of VR environments for improving spatial comprehension and potentially reducing surgical complications 10 . Despite these advances, widespread clinical adoption of VR in clinical practice remains limited, largely due to the significant manual effort required to create accurate and detailed anatomical reconstructions. Manual segmentation of structures from CT scans is time-consuming, labor-intensive, and requires expert personnel. Automated artificial intelligence (AI)-driven segmentation technologies offer promising solutions to overcome these limitations. By quickly and accurately identifying critical anatomical structures, segmentation significantly reduces model preparation time and improves the fidelity of anatomical representation in VR environments 11 12 . However, there is currently limited empirical evidence directly linking AI-assisted VR planning to measurable improvements in surgical outcomes, particularly in the context of complex chest wall resections and reconstructions 8 9 .

While VR and AI each hold considerable promises, their combined potential in this challenging surgical domain remains largely unexplored. This study aims to address this gap by retrospectively evaluating interdisciplinary, collaborative VR planning enhanced by automated AI segmentation in relation to actual surgical results. Specifically, it seeks to quantitatively compare planned resection margins, defect dimensions, and reconstruction strategies predicted by VR planning with those documented in operative and pathology reports. By conducting this analysis, the study aims to estimate the clinical utility of AI-enhanced collaborative VR planning and identify its potential impact on surgical precision and interdisciplinary communication and operative strategy. Ultimately, the goal of this study is to establish a streamlined workflow using state-of-the-art technology to optimize surgical planning for a potential future improvement of patient outcomes in the complex field of chest wall reconstruction.

## Material and Methods

This study was conducted in accordance with the principles of the Declaration of Helsinki. Ethical approval was granted by the Ethics Committee of the Medical Faculty of the University of Bonn (approval number: 2024–316-BO). As this was a retrospective evaluation of clinical data that did not involve any intervention or have any influence on clinical treatment decisions, individual patient consent was not required. All data were anonymized and handled in compliance with data privacy regulations.

### Study Design and Patient Selection

This retrospective analysis included eight exemplary cases involving complex chest wall resections and reconstruction performed at University Hospital Bonn. Cases were selected based on the availability of complete preoperative and postoperative imaging (CT scans), detailed operative reports, and comprehensive pathology findings. Inclusion criteria were:

1. patients undergoing resection of a primary or metastatic chest wall tumor;
2. availability of high-resolution preoperative CT scans suitable for segmentation;
3. complete operative reports detailing the resection margins and reconstruction technique;
4. pathology reports confirming the tumor type, stage, and resection margin status.

Exclusion criteria were cases with incomplete access to preoperative imaging or medical documentation, and cases where the preoperative CT scan quality was insufficient for accurate segmentation.

### VR System and Workflow

Virtual reality planning was conducted using the Medical Imaging XR software developed by Medicalholodeck AG (Zurich, Switzerland), deployed on-premise at the University Hospital Bonn. The system was directly integrated with the hospital’s Picture Archiving and Communication System (PACS), enabling secure access to preoperative imaging data. The central rendering server was a Supermicro (San José, CA, USA) GPU server equipped with four NVIDIA A40 GPUs (NVIDIA Corporation, Santa Clara, CA, USA), allowing simultaneous support for up to eight concurrent users through GPU virtualization. VR content was streamed wirelessly to Meta Quest 3 head-mounted displays (HMD) (Meta Platforms Inc., Menlo Park, CA, USA). Medical Imaging XR enables collaborative, multi-user sessions across multiple physical locations, supported by integrated audio communication, allowing for real-time discussion and shared visualization of the 3D reconstructed anatomy. Users can interact with the 3D anatomy through handheld controllers, enabling rotation, zoom, and cross-sectional views of the anatomy.

Automated segmentation of anatomical structures was performed using the open-source model weights from TotalSegmentator, developed by the Department of Biomedical Imaging at Basel University Hospital, Switzerland. This tool provided segmentation of major anatomical structures such as bones, major vessels, and organs but did not include tumor segmentation, which was manually delineated by the surgical team within the VR environment. The generated segmentation masks were directly integrated into the VR volume-rendering environment, enhancing visualization and interaction.

### Interdisciplinary VR Planning

Each case was reviewed collaboratively by an interdisciplinary team consisting of a specialist thoracic surgeon, a specialist oncological orthopedic surgeon, and a specialist plastic/reconstructive surgeon. The team used the Medical Imaging XR toolset within the VR environment to perform the following steps:

1. Tumor and Anatomical Landmark Identification: The team identified and virtually marked the tumor boundaries, critical anatomical landmarks (e.g., chest wall vessels, nerves, pleura), and potential resection planes.
2. Resection Margin Planning: The team collaboratively planned the resection margins according to the preoperatively known or unknown histology aiming for complete tumor removal and while considering the need to preserve critical structures.
3. Defect Dimension Estimation: The team estimated the dimensions of the resulting chest wall defect following resection.
4. Reconstruction Strategy Planning: The team discussed and planned the reconstruction strategy and considered the size and location of the defect, the availability of autologous tissues, and the potential need for prosthetic reconstruction and soft tissue coverage.
5. Documentation of Planned Approach: All planning steps, including resection margins, defect size, and reconstruction strategies were documented with screenshots and annotations in the Medical Imaging XR software.

### Quantitative Comparative Analysis

The following parameters were quantitatively compared between the VR-planned approach and actual surgical outcomes:

- Defect Dimensions: The dimensions of the chest wall defect (length, width, depth) as predicted in the VR environment were compared to the actual defect dimensions measured on postoperative CT scans.
- Reconstruction Technique: The planned reconstruction technique (as documented in the VR environment) was compared to the actual reconstruction technique used during surgery.

Statistical analysis was performed using Excel (Microsoft, Redmond, WA, USA).

### Usability Assessment

After each VR-planning session, the team completed the 26-item User-Experience Questionnaire (UEQ) (7-point semantic differential scale, −3 to +3). Following the official UEQ scoring protocol we calculated the six-dimension means – Attractiveness, Perspicuity, Efficiency, Dependability, Stimulation and Novelty – and the derived Pragmatic and Hedonic Quality indices 13 . Scale means were interpreted with the UEQ benchmark (positive > +0.8, neutral −0.8 …+0.8, negative < −0.8).

### Planning Workspace Perception

Immediately after each VR-planning session, participants completed an eight-item questionnaire developed for this study to gauge visual fidelity, realism, and interaction quality of the planning workspace. Statements were rated on a six-point Likert scale (1 = “strongly disagree”, 6 = “strongly agree”), with higher values indicating a more favorable perception.

### Cybersickness Assessment

Simulator-related discomfort was measured with the 16-item Simulator-Sickness Questionnaire (SSQ) completed before (Pre) and immediately after (Post) each VR session. Item ratings (0 = none – 3 = severe) were converted to the three standard factors – Nausea, Oculomotor, Disorientation – and to the Total SSQ score using the original Kennedy weighting (factor means × 9.54/7.58/13.92; total mean × 3.74).

A ΔSSQ value (Post – Pre) was calculated for each participant; positive values indicate a symptom increase. Group means were compared with commonly cited safety bands (negligible < 5, minimal 5–10, significant 10–15, concerning 15–20) 14 .

## Results

We retrospectively analyzed eight cases who underwent chest wall resection and reconstruction for malignant disease in our institution since 2015. Case characteristics are summarized in Table 1 . 50% of the cases were male and the other half were female, with a median age of 52 years (17–65). Five out of eight cases were treated for sarcoma.

**Table:** **Table 1** Case characteristics.

Case	Gender	Age	Diagnosis
1	Male	17	Ewing sarcoma
2	Male	62	Metastasis of a cholangiocarcinoma
3	Male	62	Metastasis of a cholangiocarcinoma
4	Female	75	Undifferentiated sarcoma
5	Male	58	Chondrosarcoma
6	Female	61	Basal cell carcinoma
7	Female	28	Chondrosarcoma
8	Female	56	Chondrosarcoma

**Fig. 1:**
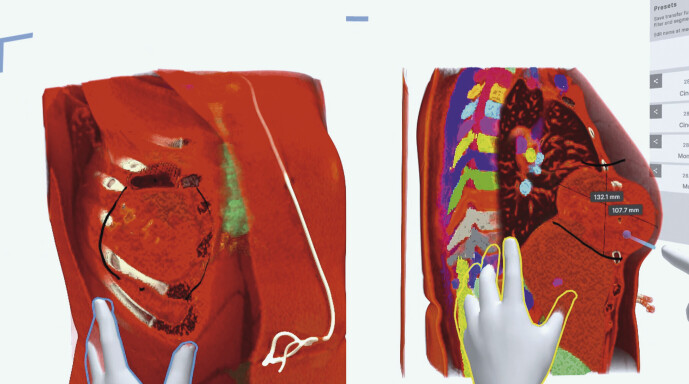
VR-reconstruction of a CT-scan showing a chest wall tumor with interactive measurement tools used in the interdisciplinary retrospective discussion.

Fig. 1 shows a participant’s HMD view: color-coded masks delineate the automatically segmented ribs, lungs, costal cartilage, liver, and other key structures, while the remaining unsegmented tissue is rendered more opaque to sharpen contrast and preserve overall spatial orientation. Because the tumor is not yet included in the auto segmentation pipeline, it inherits the background (unsegmented tissue) coloring, visually highlighting a current limitation of the workflow.

Table 2 compares the actual surgical procedures and reconstruction with those planned using VR-based simulation. Seven out of eight cases had complete tumor resection. In one case, the pathology report revealed microscopic tumor infiltration at the rib bone margins, despite the surgeon’s macroscopic impression of complete resection. In three cases (37.5%), the actual resection exceeded the extent predicted by VR planning. In four cases (50%), the extent of VR resection planning exceeded the actual resection extent (by a maximum of 24%), while in one case (12.5%) the VR planning exceeded the extent of actual resection by 332%. Discrepancies between actual and VR-predicted chest wall reconstruction occurred in two patients, specifically regarding the need for chest wall reconstruction requiring a mesh graft.

**Table:** **Table 2** Comparison between actual resection and virtual resection after VR reconstruction.

Case	Tumor location	Actual resection size (cm²)	Actual chest wall reconstruction	VR resection size (cm²)	VR chest wall reconstruction	Difference (cm²)	Difference in Percent
1	4–6. ribs right	209.3	Bio-patch+ Latissimus dorsi flap surgery	224	Patch+ Latissimus dorsi flap surgery	14.7	7%
2	Soft tissue chest	89.3	Fortiva-Patch+ Latissimus dorsi flap surgery	100	Latissimus dorsi flap surgery	10.7	12%
3	5. rib right	8.1	Vicryl patch	35	Patch	26.9	332%
4	Sternum	89.3	Ultrapro patch	100	Patch	10.7	12%
5	7. rib left	48,2	Fortiva patch	60	No reconstruction needed	11.8	24%
6	Soft tissue sternum	156	Bio-patch	40	Patch	−116	−74%
7	11–12. ribs right	145	Ultrapro patch	96	Patch	−49	−34%
8	Sternum	162,5	Bio-patch	36	Patch	−126,5	−78%

**Fig. 2:**
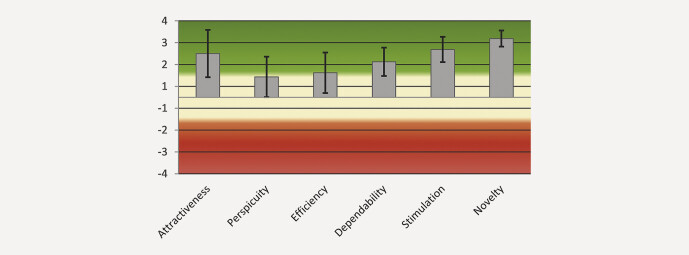
Graphic results for the User-Experience Questionnaire (UEQ: green = positive, yellow = neutral, red = negative response).

The UEQ does not produce an overall score for the user experience but enables an interpretation of the means of the scales in Fig. 2 . The mean UEQ scales and variance were as follows: Attractiveness = 2.000 (1.22); Perspicuity = 0.938 (0.89); Efficiency = 1.125 (0.90); Dependability = 1.625 (0.44); Stimulation = 2.188 (0.35); Novelty = 2.688 (0.14).

**Fig. 3:**
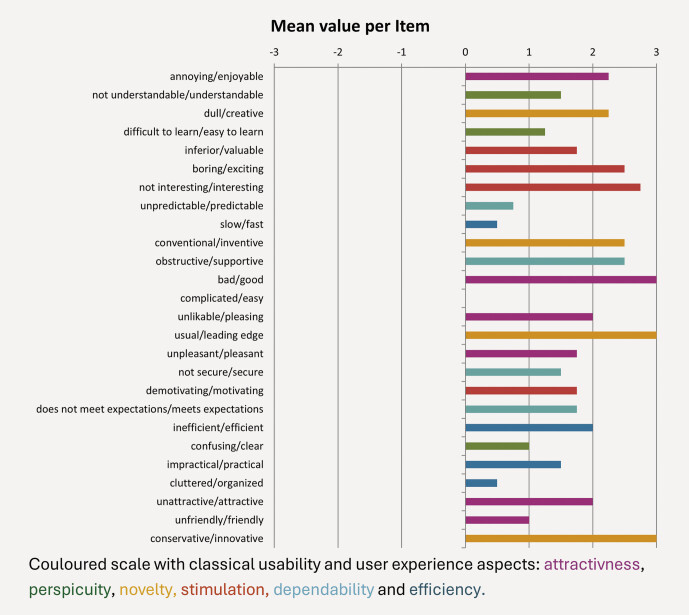
Mean value per UEQ item.

The values for the single items are listed to permit the detection of outliers in the evaluations in Fig. 3 .

**Fig. 4:**
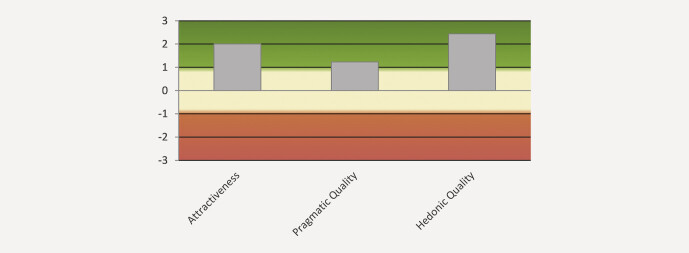
Calculation of the mean of the three pragmatic and hedonic quality aspects.

The scales of the UEQ can be grouped into pragmatic quality (Perspicuity, Efficiency, Dependability) and hedonic quality (Stimulation, Originality). Pragmatic quality describes task-related quality aspects whereas hedonic quality covers non-task-related quality aspects. In Fig. 4 , the means of the three pragmatic and hedonic quality aspects are calculated. Attractiveness mean score was 2.00 as described earlier, pragmatic quality was rated as 1.23 and hedonic quality as 2.44.

**Fig. 5:**
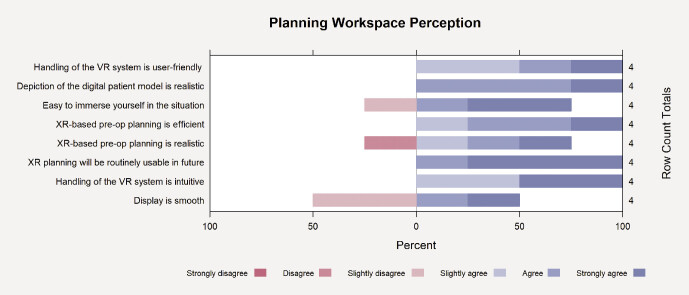
Visualization of the item-by-item response distribution for the planning workspace perception questions on a six-point Likert scale.

For the eight analyzed statements on planning workspace perception, the four participants provided an overall mean rating of 4.9 ± 0.7 on the 1-to-6 scale. Individual participant means ranged from 4.1 to 5.8. Item averages ranged from 4.3 (“display is smooth” and “XR-based pre-op planning is realistic”) to 5.8 (“XR planning will be routinely usable in future”). Of the 32 ratings recorded (4 participants × 8 items), 22 responses (69%) were “5” or “6”, 28 (88%) were at least “4”, and none fell below “2”. Fig. 5 visualizes the item-by-item response distribution for the planning workspace perception questions on a six-point Likert scale.

Overall, the weighted SSQ values were low. The mean total SSQ was 2.0 ± 1.6 at baseline (Pre) and 3.0 ± 1.3 after VR exposure (Post), giving a mean ΔSSQ of +1.0 ± 1.6. Both group means fall in the negligible band (< 5).

Factor means changed only slightly (Pre → Post): Nausea 0.4 → 1.2, Oculomotor 4.9 → 6.5, and Disorientation 3.9 → 4.8. For Oculomotor, two participants’ Pre scores were within the 5–10 minimal band, and one participant’s Post score was in the 10–15 significant band; all other factor and total scores remained below 5 throughout.

## Discussion

Chest wall resection and reconstruction in oncologic surgery remains a complex surgical task, particularly for extensive and anatomically complex tumor locations. Especially sarcoma surgery requires close interdisciplinary collaboration between thoracic surgeons, orthopedic surgeons, and plastic surgeons 15 . In addition to interdisciplinary decision-making on the sequence of multimodal treatment, precise preoperative planning, primarily regarding the extent of resection and resection margins, is essential when deciding on an effective reconstruction strategy. Especially in reconstruction strategies that include more elaborate prosthetic approaches, e.g., biological patch material from acellular porcine dermal collagen matrix or 3D-printed implants, pinpoint planning is important as certain approaches involve the use of expensive materials 16 17 .

Previous studies have shown the benefits of immersive virtual reality in planning chest wall surgery 8 . Whereas Thumerel et al. showed that VR planning improved accuracy compared to conventional 2D-CT scans, our group has demonstrated the feasibility and potential of a collaborative VR-planning tool in previous exemplary case series using immersive real-time volume-rendering to support surgical decision-making.

Given that multidisciplinary approaches have been shown to have beneficial outcomes for chest wall resection and reconstruction, our study seeks to show that multidisciplinary, AI-segmented, real-time rendered VR planning is a convenient tool for multidisciplinary teams 18 19 . Our findings show that in 3 out of 8 cases (37.5%), the intraoperative resection was more extensive than predicted by the VR simulation. The discrepancies primarily involved unexpected tumor infiltration into adjacent tissues, which was underestimated or not visible in the virtual planning setting. This shows a limitation of preoperative imaging that cannot be eliminated even with immersive visualization techniques; so far, intraoperative assessment and frozen section analysis remains unmatched. In a comparable study simulating complex 3D osseous tumor resections in plastic pelvis models, Cartiaux et al. demonstrated that experienced surgeons working under ideal conditions could achieve good surgical margins in only 52% of cases, highlighting the same challenge we encountered 20 . Therefore, we determined that an overestimation of the surgical extent by up to 24% in 4 of 8 cases was acceptable. In 1 of 8 cases (case 3), the VR-planning group greatly overestimated the extent of the necessary resection. This can be explained by the fact that the study group probably retrospectively assumed a fundamentally different oncological scenario and therefore overestimated the surgical margins. These results suggest that while VR simulation provides valuable support in surgical planning, particularly in enhancing spatial orientation and understanding anatomical relationships, it may underestimate the true extent of tumor invasion. This limitation is likely due to the dependence on imaging resolution and the difficulty in detecting microscopic or subtle signs of disease which only become apparent during surgical exploration. In conclusion, VR-based simulation is a promising tool for preoperative planning of chest wall resections but it should be considered a complementary aid rather than a definitive predictor of surgical extent. Surgeons must remain prepared to adapt intraoperatively when the actual findings exceed the scope of preoperative simulations. Further refinements in imaging integration and VR modeling may help bridge this gap in the future.

Assuming that values > 0.8 represent a positive evaluation in the UEQ, the mean scores for Attractiveness and Stimulation of 2 and higher and Novelty, which had a score of 2.688, show that the technique using VR planning with real-time rendering and AI-enhanced segmentation was adopted very well by the participating surgeons. The best assessed single items rated the approach as innovative and leading edge. The upper-level grouping of items assessing pragmatic quality was still evaluated as positive but less so than the hedonic quality. This might be consistent with the underestimation of the extent of resection where the quality of the segmentation of tumor-adjacent structures in VR reconstruction still requires improvement. Enabling precise and reliable auto segmentation of tumor tissue itself would make the assessment of resection margins significantly more user-independent and thus also more reliable. This would also have a positive effect on the subjective perception of users.

Participants rated the immersive VR planning environment positively across several dimensions. The custom planning workspace questionnaire yielded a mean score of 4.9 ± 0.7 on a 6-point Likert scale, suggesting a generally favorable perception of realism, visual fidelity, and interactive usability. The majority of responses (88%) were ≥ 4, and none fell below 2, indicating that user experience was consistently acceptable to very good. Especially high ratings were given for the statements related to future clinical applicability, implying a promising outlook for the routine integration of such systems. These findings complement the UEQ results, where particularly hedonic qualities such as Stimulation (2.19) and Novelty (2.69) stood out, reflecting not only usability but also the innovative appeal of the system.

In terms of tolerability, the Simulator Sickness Questionnaire (SSQ) revealed only minor increases in symptoms after VR use (ΔSSQ mean: +1.0 ± 1.6), with all group means remaining in the “negligible” range (< 5). While two participants already reported mild oculomotor symptoms prior to the session and one showed a post-session value within the “significant” range (10–15), these effects did not interfere with the planning sessions, and no participant had to abort the session. It should be noted, however, that intermittent technical issues, specifically occasional display jittering, were reported during some sessions, which may have contributed to discomfort or negative perceptions. These issues are addressed in ongoing system updates and are expected to be resolved in future. Taken together, the results suggest that the VR system was generally well tolerated and functionally viable for interdisciplinary surgical planning, although technical refinements remain important for a broader clinical integration.

A probable next step would be the implementation of mixed reality into preoperative surgical multidisciplinary planning for large chest wall malignancies using a holographic overlay of real-time rendered 3D-reconstructed imaging with video pass-through head-mounted displays as previously described by Arensmeyer et al. 21 .

Limitations of this study include its retrospective nature, and the limited number of patients analyzed. It is important to mention that none of the participating surgeons were involved in the original surgeries of the retrospectively analyzed patients, thereby eliminating the risk of bias due to prior knowledge or recognition of individual cases, a concern noted in previous studies 7 . To further validate the safety and effectiveness of this technology and the patient-centered multidisciplinary approach for preoperative planning and decision-making, prospective and larger multicenter studies with larger patient cohorts are needed. Future research should also include the evaluation of clinical endpoints of patient outcomes to determine the impact on patient care.

Another prospect could be the use of VR planning not only by experienced surgeons but as a simulation exercise for the purpose of training residents or trainees. The success of simulation training could be immediately controlled using a library of old cases as chest wall surgery remains a rare and demanding procedure. VR-based educational programs with collaborative real-time concepts have been shown to be technically feasible and generally accepted by medical students 22 .
